# Leptomeningeal Masses or Masquerades: A Spectrum of Diseases with Leptomeningeal Enhancement and their Mimics

**DOI:** 10.2174/0115734056340774241227080230

**Published:** 2025-01-03

**Authors:** Praveen M Yogendra, Oliver James Nickalls, Chi Long Ho

**Affiliations:** 1 Department of Radiology, Sengkang General Hospital, Singapore

**Keywords:** Leptomeningeal enhancement, Leptomeningeal mass, Leptomeningeal disease spectrum, Neoplastic conditions, MRI, Radiology

## Abstract

**Background:**

Leptomeningeal enhancement, visible on MRI, can indicate a variety of diseases, both neoplastic and non-neoplastic.

**Objective:**

This comprehensive pictorial review aims to equip radiologists and trainees with a thorough understanding of the diverse imaging presentations of leptomeningeal disease.

**Methods:**

Drawing from a retrospective analysis of MRI scans conducted between 1 January 2008 and 30 September 2022, at two tertiary teaching hospitals in Singapore, this review covers a wide range of conditions.

**Results:**

The main neoplastic conditions discussed include leptomeningeal carcinomatosis, myelomatosis, schwannoma, CNS lymphoma, and pineal region tumors. Additionally, the review addresses non-neoplastic enhancements such as meningoencephalitis, intracranial hypotension, cerebral ischemia/infarction, epidural lipomatosis, syringomyelia, Sturge-Weber syndrome, and subarachnoid hemorrhage.

**Conclusion:**

By highlighting the imaging features and patterns associated with these conditions, the review underscores the critical role of accurate diagnosis and timely management in improving patient outcomes. Enhanced understanding of these conditions can significantly improve patient outcomes through timely and effective therapeutic interventions.

## 1 INTRODUCTION

Leptomeningeal enhancement is an important imaging finding that can indicate a broad spectrum of neoplastic and non-neoplastic conditions, with Magnetic Resonance Imaging (MRI) serving as the key diagnostic tool. The term *leptomeninges*, derived from the Greek “leptos” (thin) and “meninx” (membrane), refers to the arachnoid and pia mater that envelop the brain and spinal cord. Based on the involved meningeal layers and enhancement patterns, conditions can be classified as leptomeningeal or pachymeningeal [1]. Recognizing specific imaging patterns, such as diffuse, nodular, or gyriform enhancement, is crucial for differentiating between conditions like leptomeningeal carcinomatosis and benign processes, such as meningoencephalitis or intracranial hypotension. Malignant leptomeningeal involvement often presents with key radiological features, including irregular or nodular enhancement, associated parenchymal masses, and restricted diffusion on Diffusion-Weighted Imaging (DWI). Understanding these red flags, along with other common mimics, is essential for accurate diagnosis. This review aims to enhance the diagnostic capabilities of radiologists and trainees by presenting a comprehensive overview of both neoplastic and non-neoplastic etiologies of leptomeningeal disease, underscoring the importance of early detection and timely management to improve patient outcomes.

## 2 METHODS

A retrospective review was conducted on imaging scans acquired from patients with leptomeningeal disease who underwent magnetic resonance imaging (MRI) examinations at two tertiary teaching hospitals in Singapore. The data spanned from 1 January 2008 to 30 September 2022. For some cases, cerebrospinal fluid (CSF) analysis and cytology results were available, allowing for correlations.

## 3 CASE COLLECTION LEPTOMENINGEAL NEO-PLASMS

### 3.1 Metastatic Carcinomatosis

**Fig. (1):**
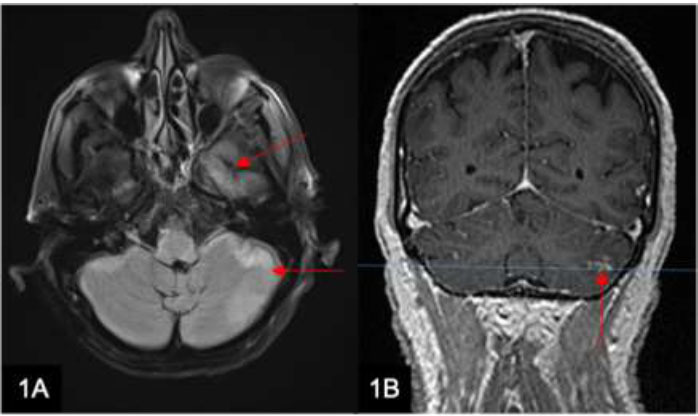
A 63-year-old man with a history of esophageal cancer presents with new-onset flaccid paraparesis and fever. Axial T2-weighted (w) FLAIR (**A**) and Coronal T1w post-contrast (**B**) MRI brain images show gyriform T2 hyperintensities along the left temporal lobe and left cerebellar hemisphere with leptomeningeal enhancement (arrows). These features are consistent with metastatic leptomeningeal carcinomatosis.

**Fig. (2):**
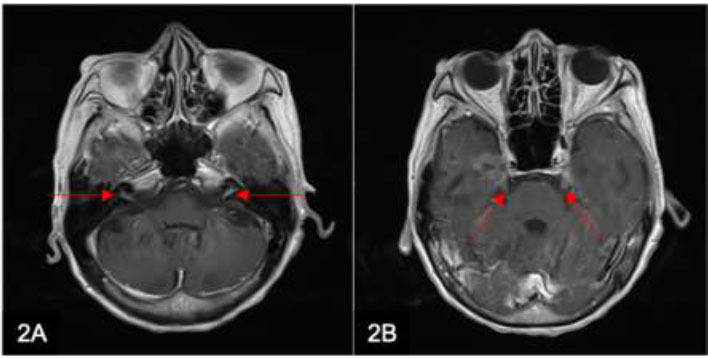
A 71-year-old woman presenting with partial seizures and a background history of lung cancer with brain metastases. Axial T1w postcontrast MRI brain images show enhancement of the bilateral 7th and 8th nerve complexes (A arrows) and bilateral 5th nerves (B arrows), consistent with leptomeningeal spread of the disease.

Leptomeningeal carcinomatosis (Fig. **1**) is a rare complication of cancer. Among leptomeningeal neoplasms, leptomeningeal metastasis is the most common. Tumor cells often metastasize *via* CSF circulation or hematogenous spread. Common primary malignancies that result in leptomeningeal spread include breast, lung, melanoma, and hematological malignancies. Metastatic spread from a primary gastrointestinal malignancy is rare (Fig. **2**). Imaging features of leptomeningeal carcinomatosis include nodular, thick, or linear enhancement of the pia, ependyma, sulci, folia, or cranial nerves [2].

### 3.2 Myelomatosis

**Fig. (3):**
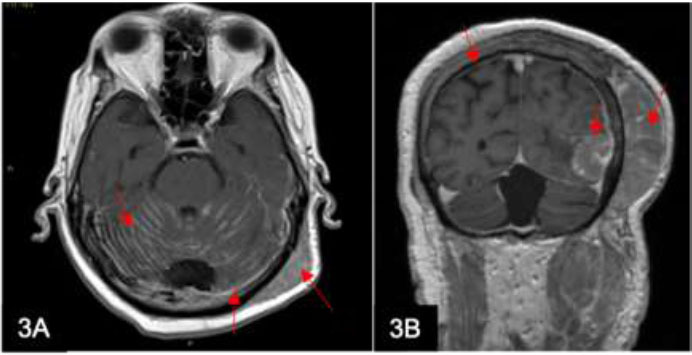
A 63-year-old man with known multiple myeloma presents with a large parieto-occipital scalp mass. Axial T1w post-contrast (**A**) and coronal T1w post-contrast (**B**) MRI show diffuse leptomeningeal (arrows) and pachymeningeal enhancement with a large heterogeneous mass involving the left temporal and parietal scalp and bones (arrows). The biopsy and CSF cytology results were positive for myeloma.

Multiple myeloma accounts for 10% of all hematological malignancies, of which 1% of patients develop leptomeningeal myelomatosis. This subgroup of patients has a very poor prognosis, with a median survival of 2 to 4 months [3]. Extramedullary myeloma (Fig. **3**) represents an aggressive form that can involve multiple sites, with leptomeningeal involvement being rare and associated with a much worse prognosis [4]. Risk factors for leptomeningeal involvement include plasmoblastic morphology, extramedullary disease, elevated lactate dehydrogenase levels, and high-risk cytogenetics [5, 6]. MRI allows for the detection of leptomeningeal enhancement; however, it is important to note that some cases may have negative MRI findings. Therefore, CSF analysis remains the gold standard for diagnosis. If cell counts are low or morphology remains inconclusive, flow cytometry can be considered [7].

### 3.3 Schwannoma

**Fig. (4):**
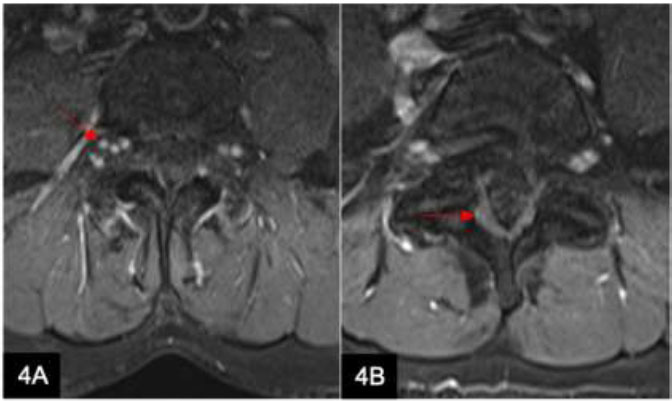
A 53-year-old woman with degenerative disc disease presents with radiculopathy. Axial T1-weighted (w) post-contrast (**A**) MRI lumbar spine image shows multiple nodular enhancements along the exiting nerve roots at the L3-L4 level, in keeping with multiple schwannomas (arrow). Axial T1w post-contrast (**B**) image shows leptomeningeal enhancement of the cauda equina (arrow).

Spinal schwannomas (Fig. **4**) are benign intradural extramedullary tumors that account for 55% of all spinal tumors. They tend to be slow-growing, encapsulated lesions seen along or attached to peripheral, cranial, or sympathetic nerves, except for optic and olfactory nerves, which lack a nerve sheath [8]. The pattern of leptomeningeal enhancement can be non-specific. Clinical correlation is required to exclude the possibility of leptomeningeal spread from a primary malignancy, though rare.

### 3.4 CNS Lymphoma

**Fig. (5):**
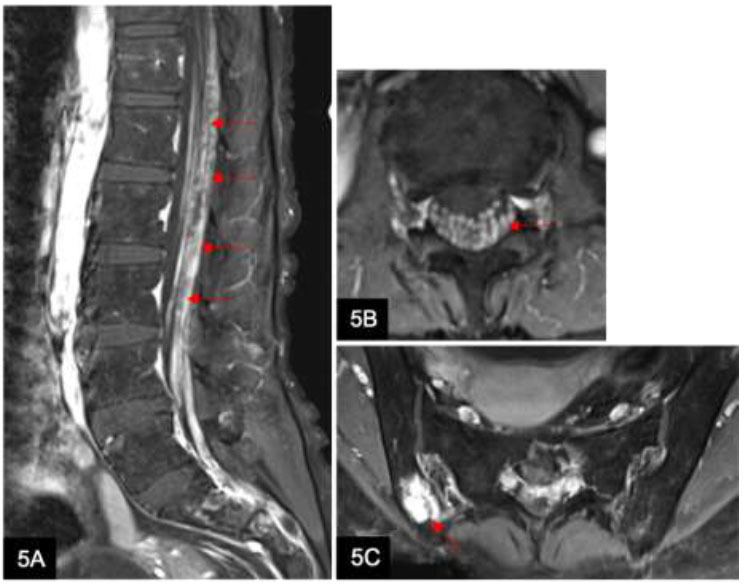
A 49-year-old woman with mantle cell lymphoma presents with bilateral lower limb weakness. Sagittal T1w post-contrast (**A**) and axial T1w post-contrast (**B**) MRI lumbar spine show extensive leptomeningeal enhancement along the cauda equina with a “sugar-coating” appearance (arrows). Axial T1w post-contrast (**C**) shows abnormal marrow infiltration (arrow) with enhancing foci in the pelvic bones. These are consistent with the leptomeningeal spread of the lymphoma in the spine.

CNS lymphoma consists of two main subtypes: primary CNS lymphoma and secondary CNS spread by systemic lymphoma (Fig. **5**). Approximately two-thirds of patients with secondary CNS lymphoma present with leptomeningeal involvement [9]. Leptomeningeal enhancement with a visible parenchymal mass seen on contrast-enhanced MRI should raise suspicion of CNS lymphoma, prompting further workup [10]. It is important to note that lumbar puncture should be avoided prior to neuroimaging as it can falsely induce dural enhancement [9].

### 3.5 Pineal Region Tumours

**Fig. (6):**
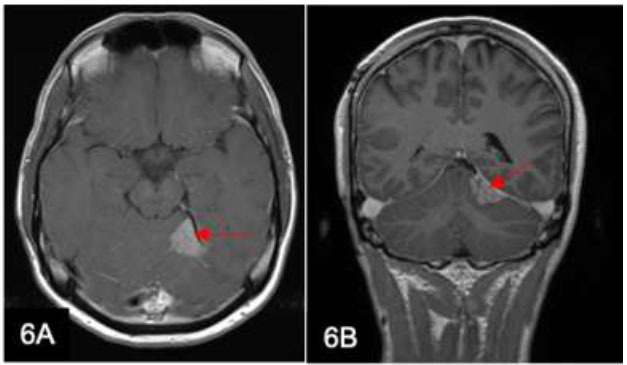
A 28-year-old man diagnosed with pineoblastoma underwent radiotherapy. Axial T1-weighted (w) post-contrast (**A**) and coronal T1w post-contrast (**B**) images show an enhancing dural-based lesion arising from the inferior surface of the left tentorium with adjacent leptomeningeal involvement (arrows). This is consistent with leptomeningeal metastasis.

Pineal region tumors constitute less than 1% of brain tumors in adults and 3-8% in children [11]. These tumors can spread along the CSF and subarachnoid spaces, including the spinal canal, resulting in drop metastases manifesting as intradural extramedullary or leptomeningeal thickening and enhancement (Fig. **6**). MRI is again the diagnostic imaging of choice for detecting leptomeningeal metastases and plays a vital role in patient surveillance. Pineal region tumors are classified into germ cell tumors (GCTs), including germinomas and non-germinomatous tumors, as well as pineal parenchymal tumors. Approximately 15% of all tumors of the pineal region are pineoblastomas [11]. These are highly malignant tumors mainly affecting children and young adults under 20 years of age. They infiltrate the pia and ependymal linings of adjacent brain structures and spread along the CSF space with seedings.

### 3.6 Bloomy-rind Sign

**Fig. (7):**
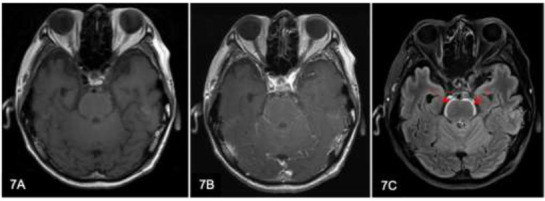
A 52-year-old man with lung adenocarcinoma underwent an MRI brain for staging purposes. Axial T1-weighted (w) (**A**) and T1w post-contrast (**B**) MRI brain images show no abnormal enhancement. Axial FLAIR (**C**) image demonstrates hyperintensity along the surface of the brainstem (arrows). This appearance is referred to as the ‘bloomy-rind sign,’ which is attributed to the spread of cancer cells to the surface of the brain in patients with lung adenocarcinoma undergoing treatment with tyrosine kinase inhibitors.

The ‘bloomy-rind sign’ describes the hyperintense signal seen on the brainstem on T2/FLAIR sequences. It has been reported in patients with lung adenocarcinoma (Fig. **7**) who are on tyrosine kinase inhibitors [12]. It has been suggested that this sign may reflect the spread of cancer cells to the leptomeninges and the superficial surface of the brain.

## 4 NON-NEOPLASTIC LEPTOMENINGEAL ENHAN-CEMENTS

### 4.1 Meningoencephalitis

**Fig. (8):**
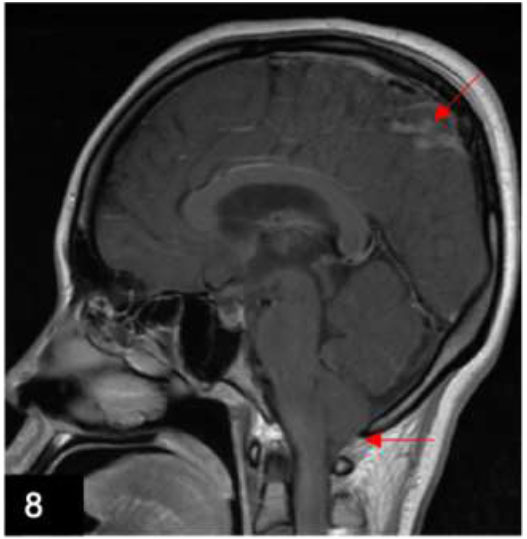
A 29-year-old woman who presented with fever, bilateral lower limb weakness, and sensory loss, which was worse on the right. Sagittal T1w post-contrast images show focal leptomeningeal enhancement at the right parietal paracentral lobule (upper arrow). Of note, low-lying cerebellar tonsils (inferior arrow) and narrowing of the interpeduncular cisterns were seen, which could be due to intracranial hypotension related to a recent lumbar puncture.

Intracranial and spinal infections result in significant morbidity and mortality. Infective causes include bacterial, viral, mycobacterial, and fungal infections (Fig. **8**). These infections increase the permeability of the meninges, allowing contrast leakage into the subarachnoid space. On imaging, bacterial and viral pathogens result in thin linear leptomeningeal enhancement, whereas mycobacterial and fungal pathogens produce thick nodular enhancement with dependent layering in the basal cisterns [2].

## 5 CONDITIONS MASQUERADING AS LEPTOMENIN-GEAL ENHANCEMENTS

### 5.1 Intracranial Hypotension

**Fig. (9):**
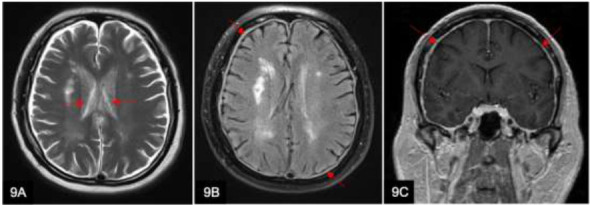
A 70-year-old woman with a past history of intracranial hemorrhage underwent ventriculostomy for hydrocephalus. Post-operative Axial T2w (**A**), axial FLAIR (**B**), and coronal T1w post-contrast MRI brain scan (**C**) show slit-like ventricles (A arrows) and diffuse pachymeningeal thickening and enhancement (B and C arrows) due to intracranial hypotension related to chronic shunting.

Common causes of intracranial hypotension include shunt over-drainage, arachnoid cyst rupture, trauma, CSF leak following lumbar puncture, or neurosurgical intervention. Approximately 10-12% of patients with ventriculoperitoneal (VP) shunts present signs of over-shunting (Fig. **9**), which include postural headache, low CSF opening pressures, and diffuse pachymeningeal enhancement [13].

### 5.2 Cerebral Ischemia/Infarction

**Fig. (10):**
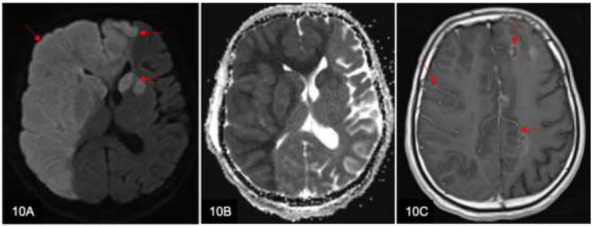
A 57-year-old man presented with seizures. Axial diffusion-weighted imaging (DWI) (**A**) and axial apparent diffusion coefficient (ADC) (**B**) MRI brain images show extensive right middle cerebral artery and bilateral anterior cerebral arteries territorial infarcts (arrows) due to occlusion of the right internal carotid artery. Axial T1w post-contrast image (**C**) shows congested leptomeningeal vessels (arrows) masquerading as leptomeningeal enhancement.

In the acute and subacute stages of cerebral infarction (Fig. **10**), leptomeningeal and pial collaterals in the areas of infarctions can masquerade as leptomeningeal enhancement. Evaluation of all MRI sequences and correlation with the clinical history could, therefore, avoid misinterpretation of the MRI findings.

### 5.3 Epidural Lipomatosis

**Fig. (11):**
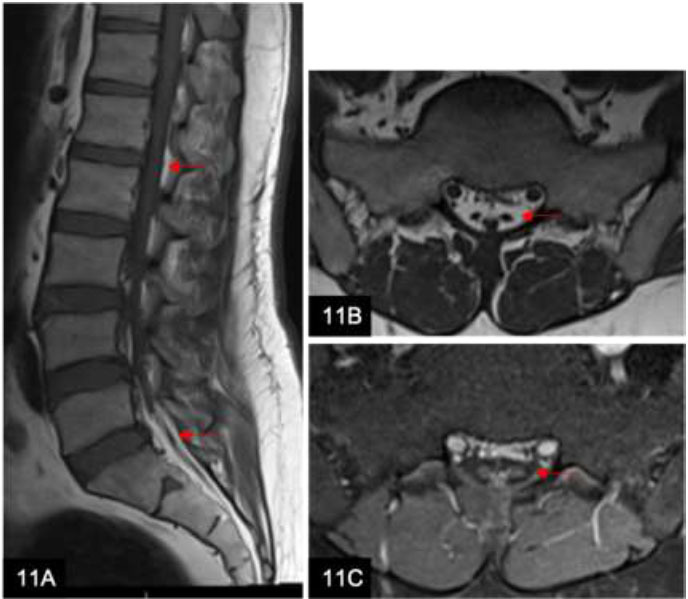
A 34-year-old man with a history of Guillain-Barré Syndrome presents with worsening lower limb weakness. Sagittal T1w (**A**), axial T1 (**B**), and axial T1w post-contrast (**C**) MRI lumbar spine scans show epidural lipomatosis with high signal intensities (A and B arrows) surrounding the cauda equina and lumbosacral nerve roots, mimicking leptomeningeal enhancement.

Epidural lipomatosis is a condition where excessive fat accumulates within the spinal epidural space, encasing the cauda equina and nerve roots (Fig. **11**). Common causes of epidural lipomatosis include long-term steroid therapy, Cushing’s syndrome, and obesity. Patients may present with non-specific symptoms such as radicular pain, weakness, and paresthesia.

### 5.4 Syringomyelia

**Fig. (12):**
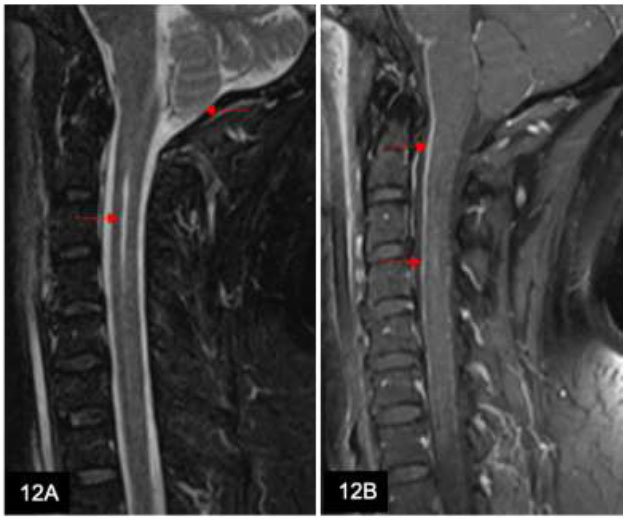
A 61-year-old man presents with acute urinary retention, fever, and hyperreflexia in all limbs. Sagittal T2w MRI of the cervical spine (**A**) shows cerebellar tonsillar descent to 5 mm beyond the foramen magnum and a long segment high T2 signal within the central spinal cord (arrows). These findings suggest a Chiari type I malformation with syringomyelia in the cervical cord. Sagittal T1w post-contrast MRI (**B**) shows congestion of the epidural venous plexus, mimicking leptomeningeal enhancement (arrows).

Syringomyelia is a condition involving the spinal cord where there is dissection through the ependymal lining of the central canal due to altered CSF dynamics. As a result, fluid-filled cavities develop within the cord parenchyma. Causes include congenital (*e.g*., Chiari malformation, myelo- meningocele, Dandy-Walker malformation) or acquired (post-traumatic, post-inflammatory, secondary to spinal cord tumor or vascular insufficiency) (Fig. **12**). On MRI, syringomyelia typically shows high T2 and low T1 signal intensities, resembling the CSF with or without flow or pulsation artifacts.

### 5.5 Sturge-weber Syndrome

**Fig. (13):**
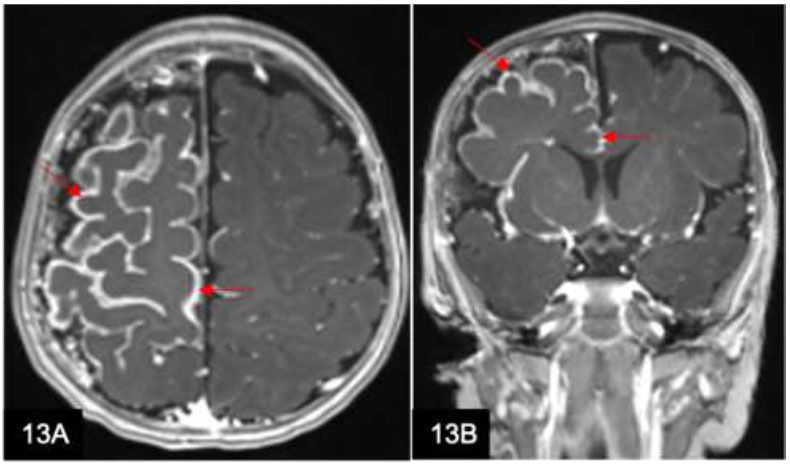
A 32-year-old man presented with discoloration of the face (so-called port-wine stains) and seizures since childhood. Axial (**A**) and coronal T1w (**B**) post-contrast MRI brain demonstrate cortical atrophy and gyriform calcification with serpentine leptomeningeal enhancement over the right cerebral hemisphere (arrows). These findings are consistent with Sturge-Weber syndrome.

Sturge-Weber syndrome (Fig. **13**) is a rare phakomatosis characterized by facial port-wine stains and pial angiomas, along with imaging features of cerebral cortical atrophy and prominent leptomeningeal enhancement secondary to congested internal cerebral veins. This manifestation of so-called ‘pial angiomatosis’ is caused by venous congestion, ischemia with infarction, and obliteration of cerebral parenchyma. The condition may be associated with enlargement of the ipsilateral choroid plexus and dilatation of the transparenchymal veins that communicate between the superficial and deep venous systems.

### 5.6 Subarachnoid Hemorrhage

**Fig. (14):**
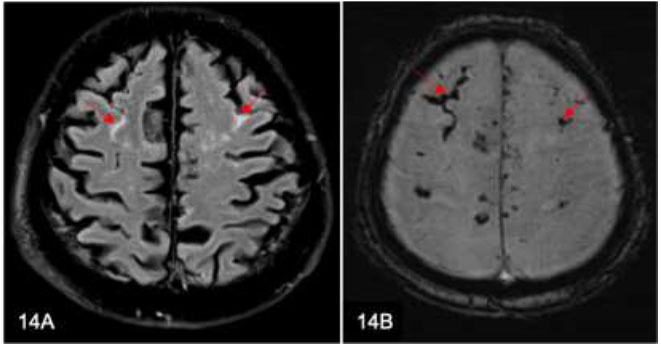
A 72-year-old man sustained an acute subarachnoid hemorrhage (SAH) following a fall incident. Axial FLAIR image (**A**) demonstrates hyperintensity along bilateral cerebral sulci, while Susceptibility-weighted imaging (**B**) demonstrates corresponding susceptibility (arrows), indicating SAH. These features masquerade as leptomeningeal disease.

Non-enhanced CT brain scans are the preferred imaging modality in emergency settings. Acute intracranial hemorrhages, including subarachnoid hemorrhage (SAH) (Fig. **14**), typically appear hyperdense on these scans. However, a false negative CT scan can occur due to severe anemia or small-volume hemorrhage. When there is a high index of suspicion for SAH, further evaluation with CT Circle of Willis or CSF analysis can be performed to check for xanthochromia. MRI is able to detect very small amounts of hemorrhage, which can appear as FLAIR hyperintensity; the corresponding susceptibility-weighted imaging (SWI) and gradient echo (GRE) images would show susceptibility. The appearance of SAH on MRI scans can, therefore masquerade as leptomeningeal disease.

## 6 DISCUSSION

Leptomeningeal enhancement encompasses a wide variety of underlying pathologies, both neoplastic and non-neoplastic, each presenting distinct imaging characteristics. The identification of these conditions on MRI is crucial for guiding appropriate diagnostic and therapeutic approaches. Leptomeningeal metastasis (LM) is a serious complication, most frequently associated with lung, breast, and melanoma cancers, with an incidence ranging between 5% and 15% [14-16]. Despite its relatively low incidence, LM often results in significant neurological morbidity due to its diffuse infiltration of the cerebrospinal fluid (CSF) space [17, 18].

In the neoplastic spectrum, leptomeningeal carcinomatosis stands out as the most common leptomeningeal malignancy. Primary malignancies such as breast cancer, lung cancer, and melanoma frequently metastasize to the leptomeninges [16, 19, 20]. Imaging features, including nodular or linear enhancement along the pia mater and cranial nerves, are critical for diagnosis [2, 14, 17]. Studies by Salehi *et al*. and Dankner *et al*. provide robust descriptions of these imaging patterns, emphasizing their diagnostic importance. However, these studies lack a detailed comparative analysis with other conditions that present similarly, potentially limiting their utility in complex differential diagnoses. Further comparative studies between leptomeningeal carcinomatosis and its mimics could enhance diagnostic accuracy in ambiguous cases. Other malignancies, such as myeloma and schwannoma, though less frequent, must also be considered as they can mimic these patterns [21].

Leptomeningeal involvement in multiple myeloma remains a rare yet aggressive complication, with poor prognosis for affected patients [3-6]. The literature outlines critical risk factors, such as plasmoblastic morphology and high-risk cytogenetics, that correlate with disease progression and diagnostic challenges. However, limitations in sample sizes within these studies reduce their generalizability, and the reliance on MRI alone may miss subtler cases. Including techniques like multiparameter flow cytometry or liquid biopsy in future studies would strengthen diagnostic precision and improve management outcomes for leptomeningeal myelomatosis.

Spinal schwannomas, although generally benign, present another diagnostic challenge due to their potential to mimic malignant leptomeningeal involvement [8, 15, 18]. The study by Nurdillah *et al*. effectively addresses these imaging patterns, providing valuable insight for differential diagnosis. However, the study’s focus on spinal schwannomas may limit its relevance in cases of cranial schwannomas, where the presentation and diagnostic considerations can differ. Expanding the study to include cranial schwannomas would enhance its diagnostic utility in a wider range of cases.

In CNS, lymphoma, particularly secondary forms, frequently involves the leptomeninges, with hallmark imaging features like “sugar-coating” enhancement patterns and associated parenchymal masses [9, 10, 14, 18]. The references offer practical guidance, especially the recommendation to avoid lumbar puncture prior to neuroimaging to prevent false enhancement findings. However, the studies would benefit from discussing alternative diagnostic protocols for situations where lumbar puncture cannot be avoided, thereby improving applicability across varied clinical settings, particularly those with limited imaging resources.

Pineal region tumors, particularly pineoblastomas in younger populations, demonstrate another route of leptomeningeal spread *via* cerebrospinal fluid dissemination [11, 12, 16]. These references underscore the importance of imaging in diagnosing and monitoring pineoblastomas, especially in younger patients. However, the studies primarily address pediatric populations, with limited discussion on atypical presentations in adults. Including cases across different age groups would enhance the generalizability of findings and offer a more complete perspective on pineal tumor behavior.

Non-neoplastic conditions, such as meningoencephalitis and intracranial hypotension, frequently present with leptomeningeal enhancements, which can be mistaken for malignancies. Bacterial and viral infections often manifest as thin or thick nodular enhancement [22]. Intracranial hypotension, often following trauma or neurosurgical interventions, may show diffuse pachymeningeal enhancement [18, 21].

Conditions like epidural lipomatosis and syringomyelia add further complexity to the differential diagnosis [18]. These are characterized by enhancement patterns that can mimic leptomeningeal disease but stem from distinct underlying processes [16].

Accurate identification of malignancy is critical in determining the appropriate treatment and management for patients presenting with leptomeningeal masses. Several imaging red flags play a pivotal role in distinguishing malignant from benign behavior.

### 6.1 Irregular or Nodular Enhancement

One of the most significant indicators of malignancy is the presence of irregular nodular enhancement patterns. Malignant masses, such as those seen in leptomeningeal carcinomatosis, often show invasive behavior, with malignant cells infiltrating the leptomeninges. This nodular enhancement is an essential imaging feature that helps differentiate malignancies from benign conditions [14, 17].

### 6.2 Thickened and Diffuse Leptomeningeal Enhancement

Thick, diffuse leptomeningeal enhancement, often associated with nodularity along the sulci or ependymal surfaces, is another hallmark of malignancy. This pattern is particularly seen in metastatic leptomeningeal disease, where extensive leptomeningeal involvement correlates with aggressive malignant behavior [15, 22].

### 6.3 Associated Parenchymal Masses

Malignant leptomeningeal lesions frequently involve both leptomeningeal and parenchymal components. In conditions such as primary CNS lymphoma or metastatic disease, the presence of combined lesions on MRI is a strong indicator of malignancy. This red flag significantly guides clinical decision-making as it suggests the potential for more extensive disease [19, 21].

### 6.4 Restricted Diffusion on DWI

Malignant leptomeningeal masses, such as those associated with CNS lymphoma, often exhibit restricted diffusion on DWI. This imaging feature is crucial in differentiating between benign processes, which typically do not show restricted diffusion, and malignant masses that demonstrate this characteristic [18].

### 6.5 Perivascular Spread and Drop Metastases

Perivascular spread and drop metastases are further red flags for malignancy, particularly in leptomeningeal metastases from tumors such as pineoblastomas. MRI often reveals evidence of CSF seeding along the spinal cord, a sign of aggressive tumor behavior. Identifying these drop metastases is crucial in assessing the extent of leptomeningeal spread [16].

These imaging features are critical in differentiating malignant from benign conditions in leptomeningeal disease. By considering these red flags on MRI, clinicians can make more informed management decisions, particularly when evaluating complex cases with overlapping imaging characteristics. Additionally, incorporating insights from studies focusing on advanced MRI in predicting malignancy could offer valuable comparative insights into malignancy detection in other settings, including leptomeningeal masses [23].

Given the broad spectrum of conditions that present with leptomeningeal enhancement, radiologists must maintain a high index of suspicion, broad differential diagnosis, and employ a multi-modal diagnostic approach, including advanced imaging and cerebrospinal fluid analysis [15, 21]. Recent advances in molecular diagnostics, such as circulating tumor DNA (ctDNA) and liquid biopsies, offer promise in detecting leptomeningeal metastasis earlier and more accurately [15, 22, 24, 25]. Additionally, studies focusing on the integration of artificial intelligence (AI) and deep learning algorithms have shown promise in the detection of LM [23, 26]. These techniques are revolutionizing the way LM is diagnosed and monitored, providing critical genetic insights and improving patient outcomes [14, 18, 27].Studies on AI and machine learning demonstrate significant potential in aiding diagnostics, although practical implementation in clinical settings is still evolving. The absence of robust validation across diverse patient populations limits the immediate applicability of these findings. Future studies focusing on protocol standardization and validation of AI techniques in routine clinical imaging could enhance the relevance and utility of these advancements.

## 7 DIAGNOSTIC AND MANAGEMENT WORKFLOW FOR LEPTOMENINGEAL MASSES

The case collection presented in this review can be further categorized into benign tumours, malignant tumours and tumour mimics (Table **1**).

**Fig. (15):**
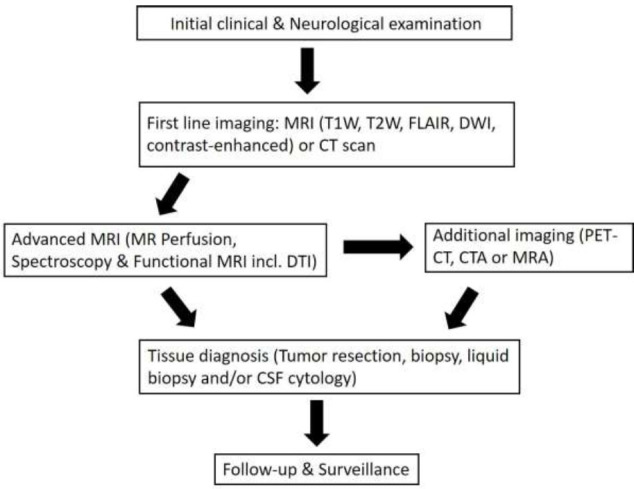
Diagnostic and management workflow for suspected leptomeningeal masses. Abbreviations: CSF: Cerebrospinal fluid, CT: Computed tomography scan, CTA: CT angiography, DTI: Diffusion tensor imaging, DWI: Diffusion-weighted imaging, MRA: Magnetic resonance angiography, PET: Positron emission tomography.

Recent advancements in imaging techniques, including new Positron Emission Tomography (PET) tracers, MR perfusion imaging, spectroscopy and functional MRI, have revolutionised diagnostic and treatment approaches for intracranial tumours [28]. Studies published in the last few years focus on new diagnostic criteria and treatment outcomes for leptomeningeal metastases or malignancies, highlighting the evolving landscape of CNS pathology [21, 29]. The evaluation of suspected cerebral masses requires a comprehensive diagnostic workflow involving both clinical assessment and a combination of imaging modalities. In our proposed diagnostic work-up, each modality offers unique strengths in identifying the nature, extent, and potential malignancy of the lesion (Fig. **15**).

### 7.1 Initial Clinical and Neurological Examination

#### 7.1.1 Patient History

• Focus on symptoms such as headache, seizures, cognitive deficits, and neurological dysfunction, which are common in mass lesions. History of cancer or systemic disease raises suspicion for metastasis or malignancy.

#### 7.1.2 Neurological Examination

Detailed assessment of motor, sensory, and cranial nerve functions helps localize the lesion.

### 7.2 First-line Imaging: Magnetic Resonance Imaging (MRI)

#### 7.2.1 Contrast-enhanced MRI

• MRI is the gold standard for evaluating cerebral masses. It provides high-resolution images of the brain structures and helps differentiate between cystic, solid, and necrotic components.

##### 7.2.1.1 T1-w Imaging (with and without Contrast)

Identifies structural details, with contrast enhancement providing insight into lesion vascularity, the integrity of the blood-brain barrier, and potential malignancy.

##### 7.2.1.2 T2-w and FLAIR Imaging

Useful in detecting edema around masses, differentiating between cystic and solid components, and highlighting surrounding inflammation.

#### 7.2.2 DWI

• Assesses cell density. Malignant masses (*e.g*., lymphoma, metastasis) often show restricted diffusion, whereas benign lesions (*e.g*., arachnoid cysts) do not.

#### 7.2.3 CT Scan

While less sensitive than MRI, a CT scan may be used in cases where MRI is contraindicated (*e.g*., claustrophobia, pacemakers, implants) or unavailable. CT is effective in detecting calcification and hemorrhage within the lesion, which are useful for specific differential diagnoses.

### 7.3 Advanced MRI in suspected cases with leptomeningeal disease: Cardiac Magnetic Resonance (CMR) in Case of Leptomeningeal Disease

• Magnetic Resonance Spectroscopy (MRS) aids in the investigation of leptomeningeal disease by offering a detailed biochemical profile of lesions, allowing for differentiation between malignancy, infection, and necrosis. By detecting key metabolites—such as elevated choline levels, which indicate cell membrane turnover in malignancy, or decreased N-acetylaspartate (NAA) levels, a marker of neuronal integrity—MRS helps clarify the nature of leptomeningeal masses. Additionally, lactate peaks identified in MRS can indicate anaerobic metabolism, often associated with necrosis or high-grade tumors, further supporting diagnostic accuracy in complex leptomeningeal conditions.

• MR perfusion imaging plays a crucial role in evaluating leptomeningeal disease by providing insights into blood flow dynamics within and around brain lesions. This imaging technique assesses tumor vascularity, distinguishing malignant leptomeningeal masses, which typically have higher perfusion due to angiogenesis, from benign lesions. By measuring relative cerebral blood volume (rCBV) and cerebral blood flow (CBF), MR perfusion also helps differentiate malignancy from infections or necrosis, as malignant lesions usually show elevated rCBV while necrotic or infectious lesions exhibit lower perfusion.

• Although primarily used for cardiac diagnostics, insights from Paolisso *et al*. (2024) demonstrate the utility of Cardiac Magnetic Resonance Imaging (CMR) in assessing mass characteristics, suggesting potential applications for evaluating leptomeningeal metastases. CMR’s ability to assess mass vascularity and spread beyond the CNS can be advantageous in diagnosing leptomeningeal involvement [23]. The study identifies features such as irregular or nodular enhancement, structural thickening, and mass effects, which are also relevant for differentiating benign from malignant leptomeningeal lesions, particularly in metastases where similar imaging patterns may be observed in the brain or spinal cord [23]. Additionally, Paolisso *et al*. developed the “CMR Mass Score,” a predictive scoring system for cardiac mass malignancies, which could inform the development of similar scoring criteria for neuroimaging to enhance malignancy detection in leptomeningeal disease [24].

• Functional MRI techniques, including Diffusion Tensor Imaging (DTI), play a crucial role in assessing the extent and impact of leptomeningeal disease on brain function. By providing detailed insights into white matter tract integrity and brain connectivity, these advanced imaging methods allow clinicians to evaluate how leptomeningeal masses may disrupt structural and functional pathways. DTI, in particular, maps the orientation and coherence of white matter fibers, helping to visualize areas affected by the disease and assess potential impacts on cognitive and motor functions. This functional imaging approach complements traditional MRI, enhancing diagnostic accuracy and guiding treatment planning by revealing the full scope of disease involvement in the brain.

### 7.4 Additional Imaging Modalities

#### 7.4.1 CT Scan

• While less sensitive than MRI, a CT scan may be used in cases where MRI is contraindicated or unavailable. CT is effective in detecting calcification and hemorrhage within the lesion, which are useful for specific differential diagnoses.

#### 7.4.2 PET

• A PET scan, especially using Fludeoxyglucose (FDG), can assess metabolic activity. Hypermetabolic activity is suggestive of malignancy, while lower uptake may indicate benign lesions.

#### 7.4.3 CT Angiography (CTA) or Magnetic Resonance Angiography (MRA)

• If vascular involvement or malformations are suspected, CTA or MRA can help to visualise the vasculature associated with the mass.

### 7.5 Tissue Diagnosis

#### 7.5.1 Tumour Resection and Biopsy

• Surgical resection or biopsy allows for direct histological examination of the tissue, providing definitive information about the tumor type, grade, and molecular characteristics. For LM, a biopsy can confirm a malignancy, identify specific tumor markers, and differentiate between primary CNS tumors and metastatic lesions from extracranial sites.

#### 7.5.2 Liquid Biopsy

• Typically performed using blood or CSF, detects ctDNA or other biomarkers. In cases where traditional biopsy is not feasible, liquid biopsy can reveal genetic mutations or alterations associated with malignancy, which aids in identifying the primary tumor site and selecting targeted therapies. This technique is minimally invasive and can also monitor tumor progression or response to treatment.

• CSF Cytology involves analyzing cerebrospinal fluid samples for the presence of malignant cells, making it a crucial diagnostic tool for detecting LM, as malignant cells frequently spread through the CSF. While CSF cytology is highly specific, its sensitivity can be limited, often necessitating multiple samples to increase diagnostic accuracy. This approach helps confirm malignancy, but the need for repeated sampling highlights the challenges in capturing malignant cells due to their often sparse distribution in the CSF.

### 7.6 Follow-Up and Surveillance

#### 7.6.1 Regular Imaging

• Serial MRI is important in monitoring lesion progression or response to treatment. MRI with contrast helps visualize recurrence or malignant transformation.

• Having a comprehensive diagnostic work-up involving MRI as the first-line modality, complemented by advanced MR and additional imaging techniques, can ensure an accurate evaluation of suspected cerebral masses. These imaging methods not only assist in differentiating between benign and malignant lesions but also play a key role in guiding treatment decisions and patient management.

## CONCLUSION

This review highlights the wide spectrum of neoplastic and non-neoplastic conditions that present with leptomeningeal enhancement and thickening, underscoring the indispensable role of Magnetic Resonance Imaging (MRI) in their diagnosis and management. As the gold standard for identifying leptomeningeal metastases, MRI plays a pivotal role in guiding both initial diagnosis and continuous patient monitoring. By recognizing critical imaging indicators—such as irregular or nodular enhancement, diffuse thickening, and associated parenchymal masses—radiologists can enhance diagnostic accuracy and effectively differentiate between malignant and benign processes. As MRI techniques and diagnostic capabilities continue to advance, it is essential for radiologists and healthcare providers to maintain expertise in interpreting these complex imaging features to ensure timely and precise diagnoses. Early detection remains paramount for enabling appropriate treatment interventions andimproving patient outcomes. In a field where prompt recognition and intervention can significantly influence prognosis, a comprehensive understanding of these MRI characteristics enhances both diagnostic precision and the quality of care delivered to patients with leptomeningeal disease. AI shows promise for improving diagnostic precision, yet challenges remain regarding their integration into routine practice. Further research aimed at validating these technologies across diverse populations will be essential in enhancing the diagnostic workflow for leptomeningeal diseases.

## Figures and Tables

**Table 1:** Summary of the key imaging features of a collection of cases with leptomeningeal masses and their mimics.

**Category**	**Condition**	**Imaging Characteristics**
**Malignant Tumors**	Leptomeningeal Carcinomatosis	Nodular, thick, or linear enhancement of pia, ependyma, sulci, folia, cranial nerves
	CNS Lymphoma	Leptomeningeal enhancement with parenchymal mass on contrast-enhanced MRI
	Pineal Region Tumors	Leptomeningeal metastases with drop metastases along the spinal canal, enhancement
**Benign Tumours**	Schwannoma	Nodular enhancement of nerve roots, can mimic leptomeningeal enhancement
	Myelomatosis	Diffuse leptomeningeal and pachymeningeal enhancement with bony involvement
**Tumour Mimics**	Intracranial Hypotension	Diffuse pachymeningeal enhancement, slit ventricles on MRI
	Epidural Lipomatosis	Fat accumulation in epidural space, high signal intensity surrounding cauda equina
	Subarachnoid Hemorrhage	FLAIR hyperintensity in cerebral sulci, corresponding susceptibility on MRI
	Syringomyelia	Congestion of epidural venous plexus
	Sturge-Weber Syndrome	Cortical atrophy, gyriform calcification with serpentine leptomeningeal enhancement
	Meningoencephalitis	Linear or nodular leptomemingeal enhancement
	Cerebral ischaemia / infarction	Congested leptomeningeal vessels

## LIST OF ABBREVIATIONS

CSF: Cerebrospinal fluid
VP: Ventriculoperitoneal
DWI: Diffusion-weighted imaging
ADC: Apparent diffusion coefficient
SAH: Subarachnoid hemorrhage
SWI: Susceptibility weighted
GRE: Gradient echo
LM: Leptomeningeal metastasis
CMR: Cardiac Magnetic Resonance
ctDNA: Circulating tumor DNA
AI: Artificial Intelligence
MRS: Magnetic Resonance Spectroscopy
NNA: N-acetylaspartate
PET: Positron Emission Tomography
FDG: Fludeoxyglucose
CTA: CT Angiography
MRA: Magnetic Resonance Angiography
